# Severe osteolysis and periprosthetic femoral fracture 45 years after acrylic hemiarthroplasty of the hip: a case report

**DOI:** 10.1186/s12891-021-04380-9

**Published:** 2021-05-25

**Authors:** Hisatoshi Ishikura, Masaki Nakamura, Kenta Matsuda, Takeyuki Tanaka, Hirotaka Kawano, Sakae Tanaka

**Affiliations:** 1 Department of Orthopaedic Surgery, University of Tokyo, 7-3-1 Hongo, Bunkyo-ku, Tokyo, 113-8655 Japan; 2 Department of Orthopaedic Surgery, Toranomon Hospital, Tokyo, Japan; 3 Department of Orthopaedic Surgery, Teikyo University, Tokyo, Japan

**Keywords:** Hip, Acrylic prosthesis, Hemiarthroplasty, Periprosthetic fracture, Osteolysis, Case report

## Abstract

**Background:**

Hip arthroplasty using acrylic prosthesis was once conducted; however, it has now been abandoned because of early breakages and wear of material. Therefore, complications or revision surgeries due to the use of acrylic prostheses are becoming rare.

**Case presentation:**

A 76-year-old woman presented with a sudden onset of severe pain in her left femur while walking. Radiographs revealed severe osteolysis and periprosthetic femoral fracture 45 years after the initial hemiarthroplasty using an acrylic prosthesis. We performed a Girdlestone resection arthroplasty by removing the prosthesis and fixing the fracture site using an intramedullary nail and metal plate. The patient was pain-free in her hip and leg 2 years and 9 months after the surgery. Although she walked with a cane or crutches, no postoperative complications were observed.

**Conclusions:**

The combined use of an intramedullary nail and plate fixation with resection arthroplasty could offer acceptable results for patients with severe osteolysis and periprosthetic fracture after femoral head replacement using an acrylic prosthesis. Our report seems relevant because it not only reminds us of the significant steps made in the development of modern total hip arthroplasty; it also highlights one of the surgical options for severe osteolysis and periprosthetic fracture of the hip.

## Background

Various materials have been tested in the history of modern hip arthroplasty. Pioneers of hip arthroplasty experimented with interpositional arthroplasty between the late 19th and early 20th centuries using various tissues, including fascia lata, skin, pig bladders, etc. [1].

In the 1920s, Smith-Petersen introduced the first mold cup made from glass, which covered the reshaped femoral head [2]. Because of its fragility, he tried other materials, including vitallium, which was made from a cobalt-chrome alloy.

During the development of hip arthroplasty, femoral head replacement using acrylic prosthesis was first introduced by the Judet brothers in 1947; their method was first reported in French and later in English [3, 4]. It was the “resection-reconstruction” operation, consisting of the excision of the femoral head and replacement with an artificial head made of polymethylmethacrylate implant with a stem [4]. The stem was designed to penetrate the lateral cortex in the subtrochanteric area.

In the 1950s, several types of acrylic hemiarthroplasty implants were also developed in Japan [5]. The Judet prosthesis was a femoral cervical supporting type, whereas the Japanese types were femoral shaft supporting types [6].

These acrylic prostheses achieved promising early results [4, 7], but reports of loosening, mechanical failures, and breakages were presented during the same period [8, 9]. Subsequently, many revision cases for these acrylic prostheses due to some complications have been reported [10–12]. Because of these poor clinical outcomes, the hip arthroplasty using the acrylic prosthesis had faded out by the mid-1970s.

We report a case of severe osteolysis and periprosthetic fracture 45 years after surgery with an acrylic femoral head prosthesis, which was treated with the combined use of an intramedullary nail and plate fixation with Girdlestone resection arthroplasty. This report is unique because it is the first reported revision surgery for the periprosthetic femoral fracture derived from the acrylic prosthesis.

## Case presentation

A 76-year-old woman (height, 149 cm; weight, 55 kg; body mass index, 24.8 kg/m^2^) suddenly experienced severe left femoral pain when walking with a T-cane. After this event, the patient was brought in a wheelchair to our hospital.

When the patient was 7 years old, she underwent several surgeries for septic arthritis and osteomyelitis of the left hip (Table 1). After that, she could live a normal life without any hip pain. However, the left hip pain gradually increased in her late 20s, and she was diagnosed with severe left hip joint destruction. Subsequently, she underwent femoral head replacement surgery with an acrylic prosthesis when she was 31 years old (45 years previously). Her hip pain was relieved for over 30 years. When she was 70 years old, she had difficulty walking without a T-cane because the left hip pain recurred, and she noted a leg length discrepancy, which gradually increased. She had not gone to the hospital before this sudden onset of left hip pain. She had a medical history of hypertension and dyslipidemia, which are controlled by oral administration.

**Table 1:** Timeline

1949	Several surgeries for septic arthritis and osteomyelitis of the left hip
1973	Hemiarthroplasty for the left hip joint destruction with acrylic prosthesis
2012	Pain in the left hip
2018	Sudden onset of severe pain in the left hip due to the periprosthetic fracture
	Combined use of an intramedullary nail and plate fixation with resection arthroplasty

**Fig. 1:**
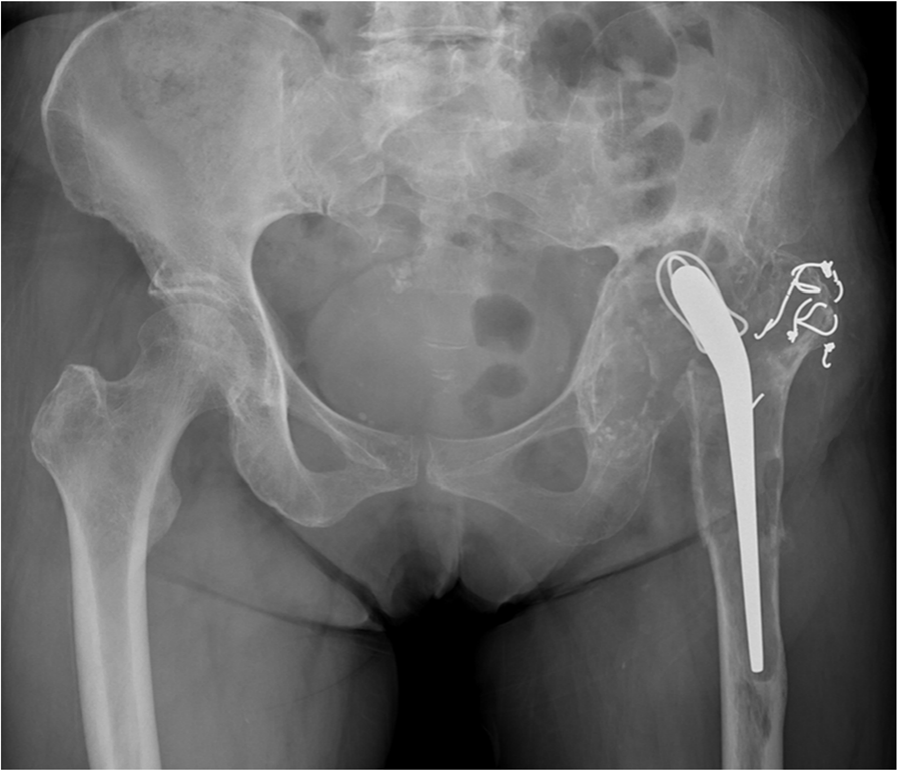
A plain anteroposterior radiograph of the bilateral hip at the patient’s first visit showing extensive osteolysis around the implant and upper migration of the left femur

**Fig. 2:**
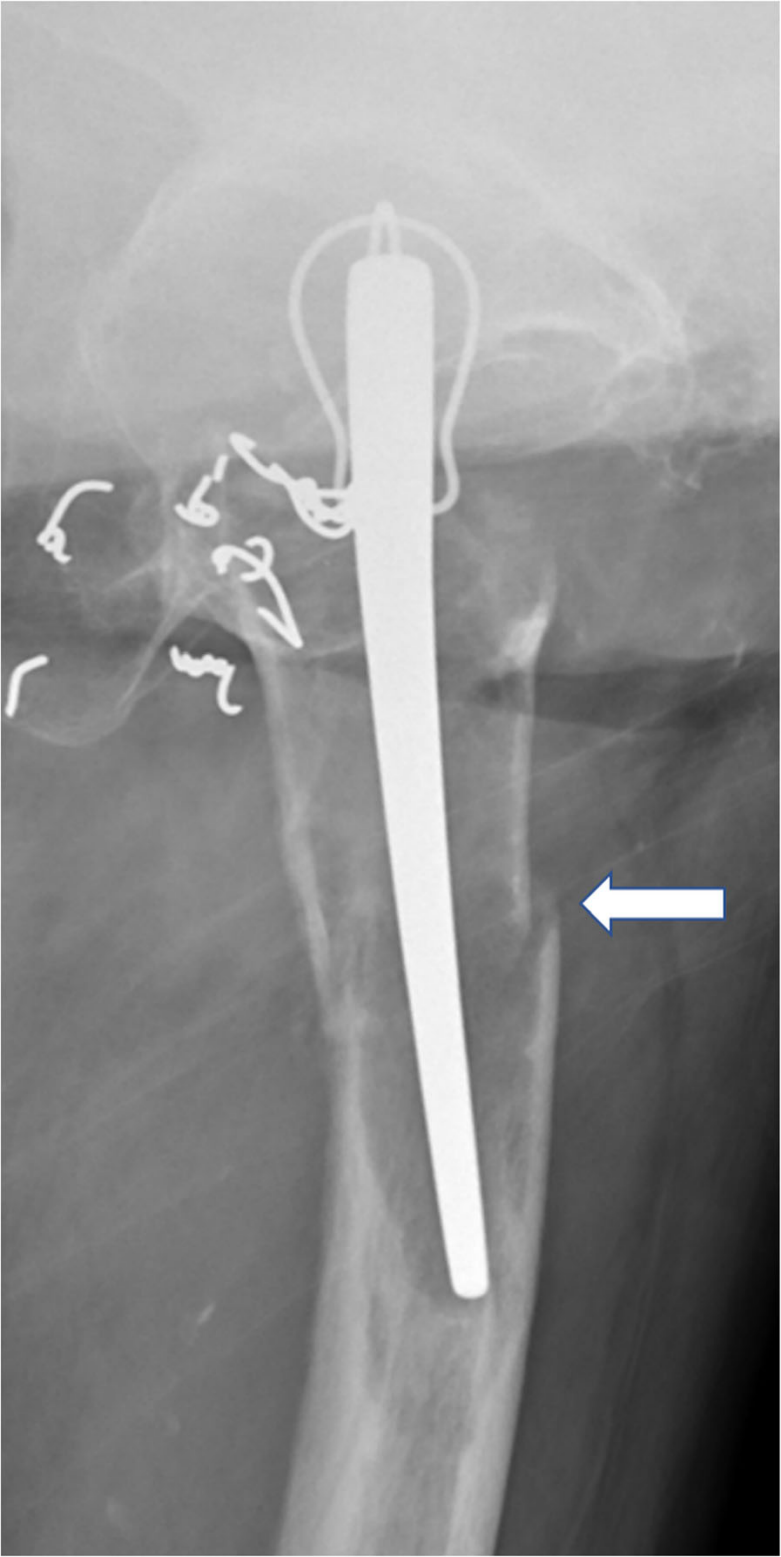
A plain lateral radiograph of the left hip at the patient’s first visit showing periprosthetic fracture of the left femur (arrow)

**Fig. 3:**
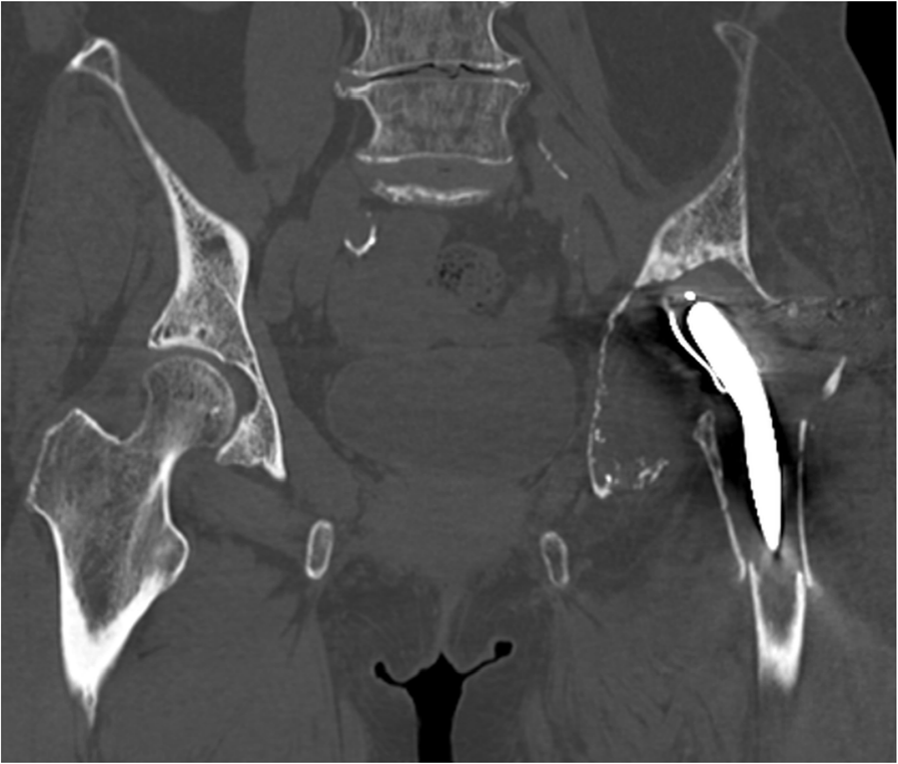
A plain computed tomography image of the bilateral hip in the coronal section showing a notably flimsy inner table of the left acetabulum. The periprosthetic fracture of the left femur can also be seen in this view

**Fig. 4:**
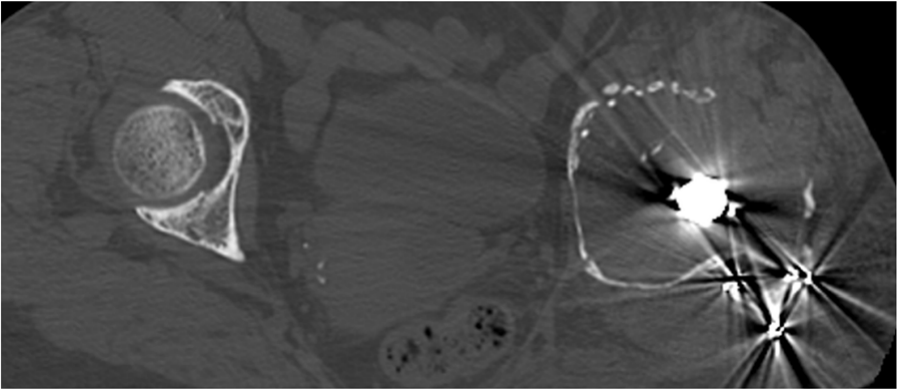
A plain computed tomography image of the bilateral hip in the axial section showing the fragmented anterior wall of the acetabulum and the extremely thin inner table

Physical examination of the left hip showed no active motion and remarkably limited passive motion due to severe hip pain. An anteroposterior radiograph showed severe bone stock loss with upper migration of the prosthesis in the acetabulum. Obvious prosthesis loosening was recognized in combination with the thinning of the femoral cortex (Fig. 1). A lateral radiograph showed a periprosthetic fracture of the femur (Fig. 2), and computed tomography demonstrated marked bone loss with a flimsy and fragmented acetabular roof (Figs. 3, 4).

We took into account several factors, including the imaging findings, patient’s age, activities of daily living before the injury, and the patient’s choice before selecting a procedure. We decided to perform a Girdlestone resection arthroplasty by removing the acrylic prosthesis and conducting internal fixation of the periprosthetic fracture.

**Fig. 5:**
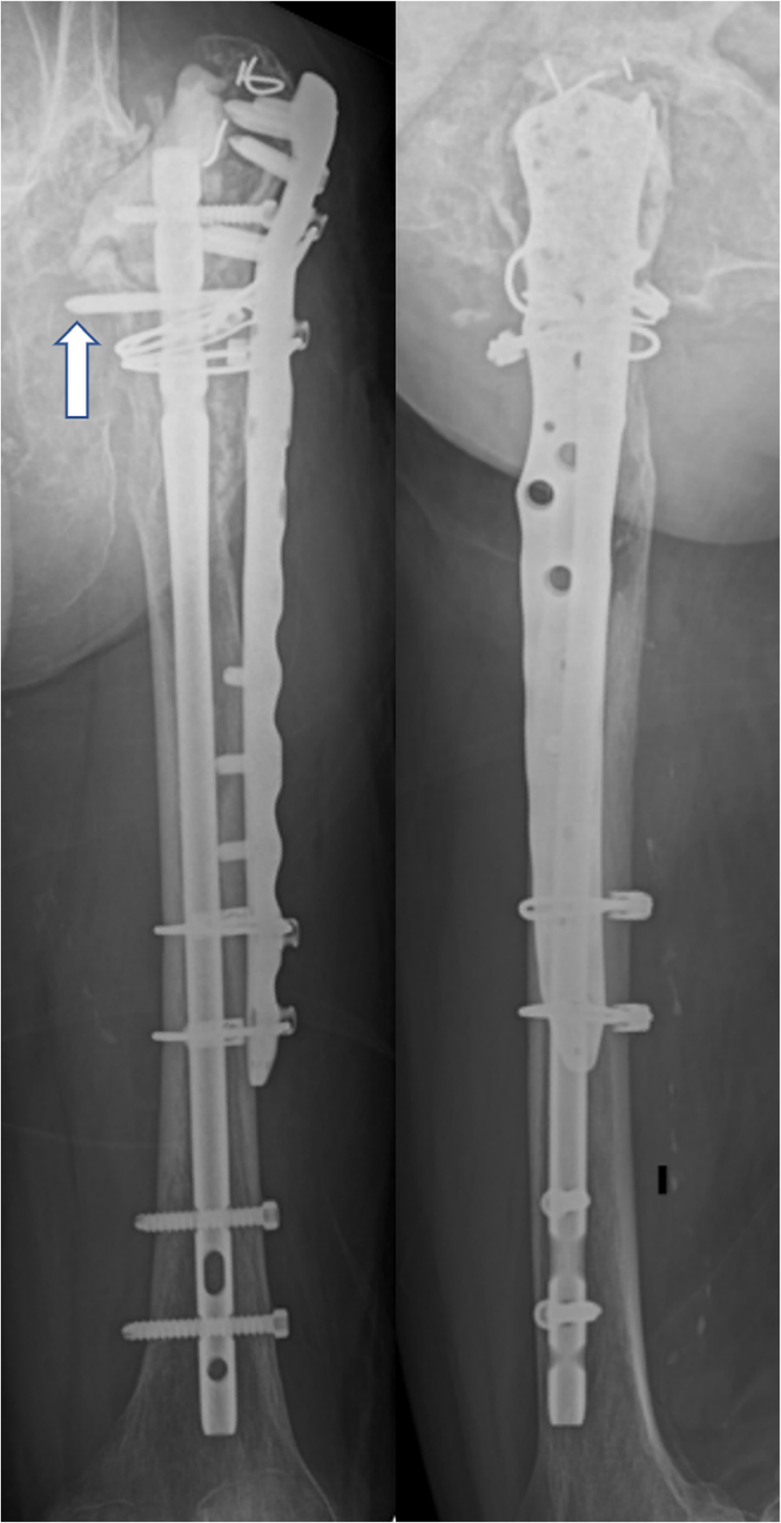
Postoperative plain radiographs of the left femur (left: anteroposterior view, right: lateral view). A locking screw of the plate was placed through the intramedullary nail to reinforce the fixation (arrow)

**Fig. 6:**
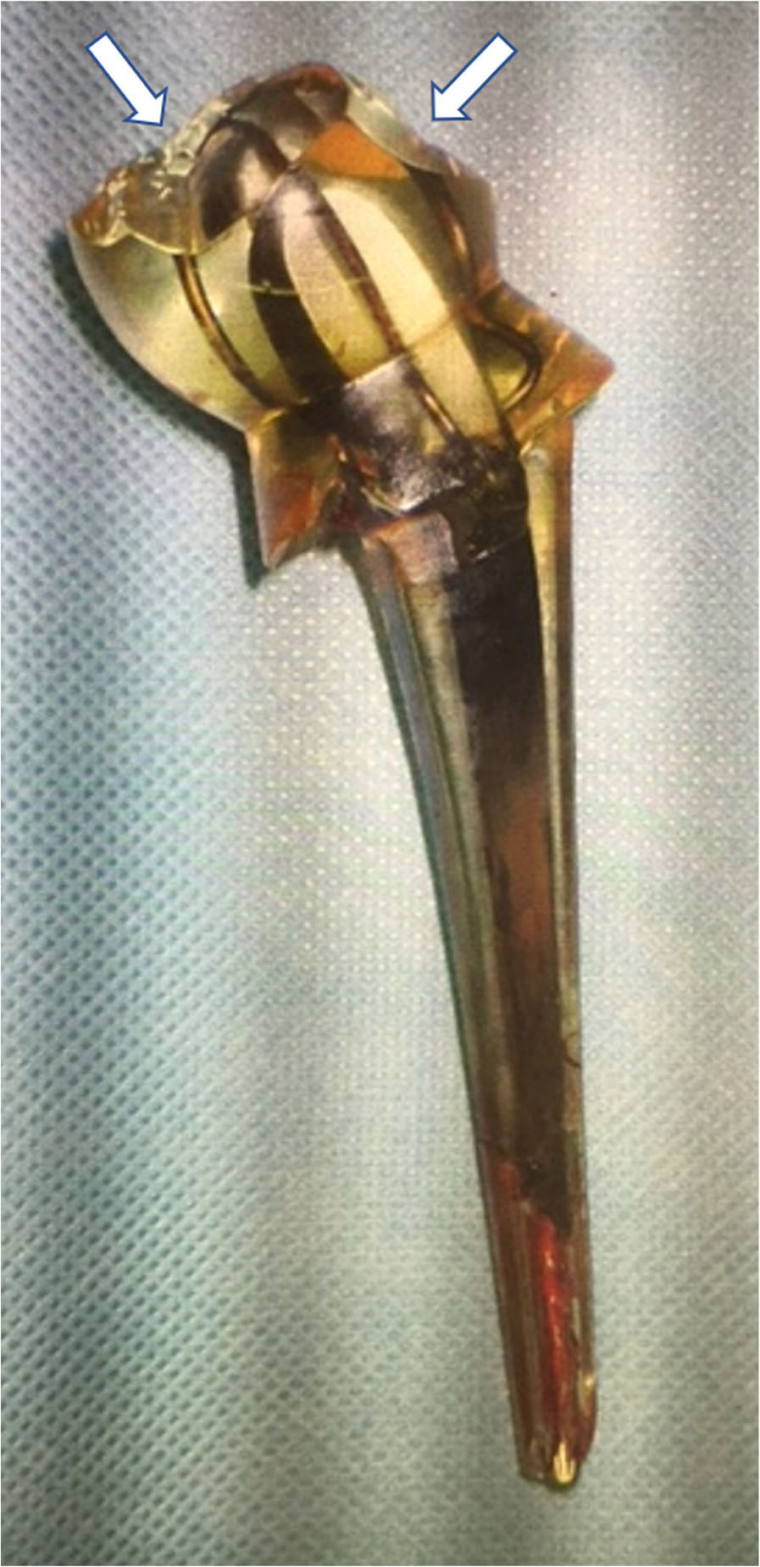
A photograph of the removed acrylic femoral head prosthesis showing remarkable wear-out in the acrylic head (arrows)

During the surgery, we recognized the severely loosened prosthesis and removed it easily by hand. There was granulation tissue formation around the implant. The femoral cortex was extremely thin, in which we could confirm the fracture site. For internal fixation, we combined an intramedullary nail (T2 femoral nail, Stryker Corporation, Kalamazoo, Michigan, USA) with plate fixation (NCB distal femur plate, Zimmer Biomet Holdings, Warsaw, Indiana, USA). In particular, a locking screw of the plate was placed through the intramedullary nail to reinforce the fixation (Fig. 5, arrow). We placed allografts around the fracture site and the proximal part of the nail to fill the defect. The top of the nail was covered with a cement block so that it would not irritate the acetabular bone or other soft tissues. Finally, several cerclage wires were placed around the plate and femur. The removed implant showed remarkable wear-out, especially in the acrylic head (Fig. 6).

Physiotherapy was started the day after the surgery. Range of motion exercise for the hip was started as soon as possible. For the first 8 weeks after the surgery, toe-touch weight-bearing was permitted. After increments in partial weight-bearing, full weight-bearing was permitted 12 weeks after the surgery.

**Fig. 7:**
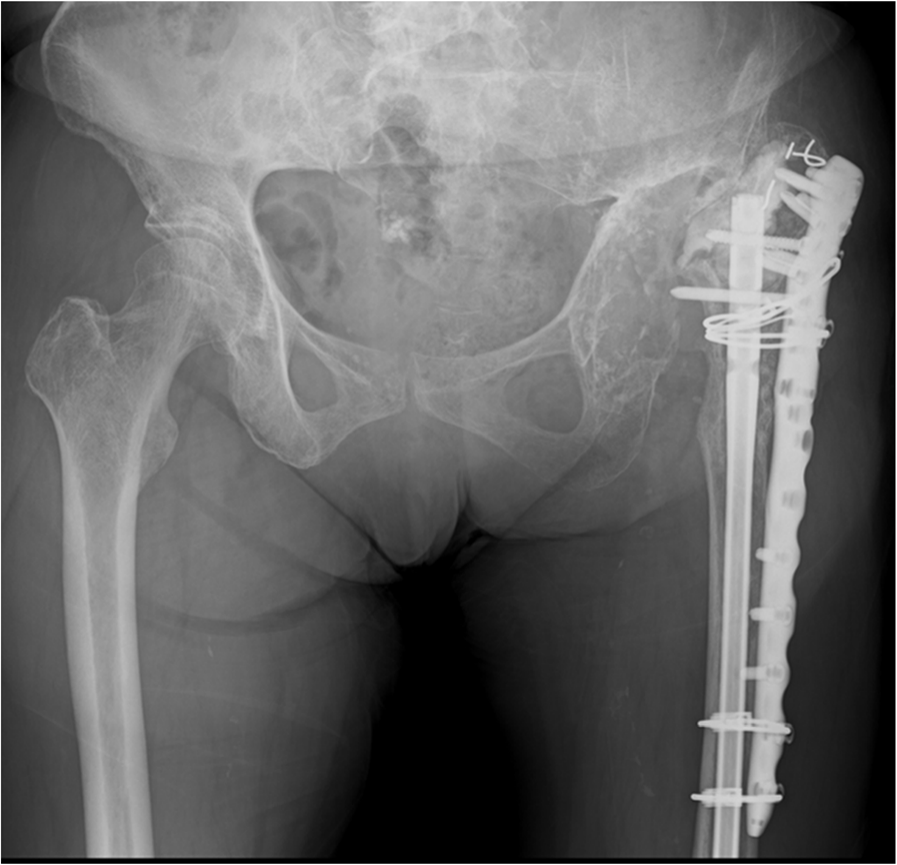
A plain anteroposterior radiograph of the bilateral hip 2 years and 9 months after the second surgery. Bone union can be observed

After 2 years and 9 months of follow-up, bone union was observed on radiography (Fig. 7). The patient could live with negligible pain in her left hip, walking with the T-cane indoors and crutches outdoors. The Harris hip score of her left hip at the last visit was 74. No unanticipated events occurred during the follow-up.

## Discussion and conclusions

Until now, few studies have described the cases of acrylic prosthesis beyond 40 years of follow-up because of its poor clinical outcomes [6, 11–13].

Uriarte et al. reported the longest follow-up (65 years) of a Judet prosthesis [13]. In that case, the implant was removed because the stem was markedly loosened and was protruding from the lateral aspect of the thigh. It also showed extensive osteolysis in the acetabulum and proximal femur.

In our present case, 45 years after initial hemiarthroplasty, severe loosening and osteolysis were recognized as in many other cases. The current case is unique because it involves a periprosthetic femoral fracture, which caused the sudden onset of severe pain.

Revision total hip arthroplasty was one of the options for this case, but it had several disadvantageous conditions.

The impaction bone grafting with metal mesh, trabecular metal augments, and antiprotrusio cages are often used to reconstruct the acetabular defect in revision total hip arthroplasty [14, 15]. We estimated that the reconstruction by the impaction bone grafting or trabecular metal augment was inadequate to provide satisfactory strength. In such conditions, the antiprotrusio cages, such as the Burch-Schneider cage, the Kerboull reinforcement device, and the GAP ring, have been used to reinforce the reconstruction [14]. Typically, the proximal part of the Burch-Scheider cage should be secured to the ilium with screws. Moreover, the distal part of the cage should be inserted into the ischium or be fixed to the ascending ischial ramus with multiple screws. However, the osteolysis extended to the ischium and ilium, and no sufficient bone was remained to fix the cage, as described in Fig. 1. In the Kerboull reinforcement device and the GAP ring, the hook of the device should be placed under the teardrop portion. Therefore, as described in Fig. 3, the thin and fragile teardrop of this case would not offer sufficient strength.

Saddle, ice-cream cone, modular prostheses, and three-dimensional (3D) print custom-made implants, which are usually needed for the reconstruction following the periacetabular tumor resection, are other choices to reconstruct the extensive acetabular bone loss [16–18]. However, the long operation time and massive bleeding remain a problem [17]. A previous study reported a high infection rate (14%) in modular hemipelvic endoprosthesis [17]. Other complications, including repeated dislocation, the breakage of screws or prostheses, were also reported in 3D print custom-made implants [18].

The femur of this case also had adverse conditions. As this case was classified as Type B3 with the Vancouver classification, using a long stem revision with mesh, impaction bone grafting, and strut allografting was conceivable [19]. These procedures for the femur were not impossible. However, the reconstruction of the femur should be conducted on the premise of the reconstruction of the acetabulum.

Overall, we estimated that the revision total hip arthroplasty by reconstructing both the acetabulum and femur for this patient was too risky to perform. Consequently, we decided to conduct the internal fixation of the femur with the Girdlestone arthroplasty.

Girdlestone resection arthroplasty is performed for patients with a severe infection of the hip joint or who cannot undergo reimplantation because of the unacceptable surgical risk, technical difficulties, or patient rejection [20]. In this patient, this procedure was chosen because of the technical difficulties and possible postoperative complications of revision total hip arthroplasty, such as infection, dislocation, and aseptic loosening of implants.

We found that the femoral bone was too fragile to be fixed with only one implant during surgery. Combining an intramedullary nail and a metal plate must have provided sufficient strength to the fracture site. This surgical procedure has been reported as a useful technique for treating segmental femoral fractures [21]. Postoperatively, she had negligible pain and no complications mentioned above. Above all, the patient was satisfied with her present condition based on our last follow-up.

There are several limitations to this report. First, due to the sample size (*n* = 1), we recognize that additional studies are needed to determine what type of treatment is best for this kind of patient. Moreover, at the first visit to our hospital, 45 years had already passed since the first surgery. Therefore, we could not specify the detailed time course of the hip joint after the first surgery.

As there have been continual improvements in hip implants, cases of severe osteolysis due to an acrylic prosthesis, as seen in this patient, will become more uncommon and possibly inexistent in the near future. However, our report seems relevant because it not only reminds us of the significant steps made in the development of modern total hip arthroplasty, it also highlights one of the surgical options for severe osteolysis and periprosthetic fracture of the hip.

## Data Availability

The datasets used and analysed during the current study are available from the corresponding author on reasonable request.
